# Targeting PI3Kδ and PI3Kγ signalling disrupts human AML survival and bone marrow stromal cell mediated protection

**DOI:** 10.18632/oncotarget.9289

**Published:** 2016-05-11

**Authors:** Genevra Pillinger, Niamh V. Loughran, Rachel E. Piddock, Manar S. Shafat, Lyubov Zaitseva, Amina Abdul-Aziz, Matthew J. Lawes, Kristian M. Bowles, Stuart A. Rushworth

**Affiliations:** ^1^ Department of Molecular Haematology, Norwich Medical School, University of East Anglia, Norwich Research Park, Norwich, NR4 7TJ, United Kingdom; ^2^ Department of Haematology, Norfolk and Norwich University Hospitals NHS Trust, Norwich, NR4 7UY, United Kingdom

**Keywords:** PI3Kδ, PI3Kγ, AML, bone marrow stromal cells, duvelisib

## Abstract

Phosphoinositide-3-kinase (PI3K) is an enzyme group, known to regulate key survival pathways in acute myeloid leukaemia (AML). It generates phosphatidylinositol-3,4,5-triphosphate, which provides a membrane docking site for protein kinaseB activation. PI3K catalytic p110 subunits are divided into 4 isoforms; α,β,δ and γ. The PI3Kδ isoform is always expressed in AML cells, whereas the frequency of PI3Kγ expression is highly variable. The functions of these individual catalytic enzymes have not been fully resolved in AML, therefore using the PI3K p110δ and p110γ-targeted inhibitor IPI-145 (duvelisib) and specific p110δ and p110γ shRNA, we analysed the role of these two p110 subunits in human AML blast survival. The results show that PI3Kδ and PI3Kγ inhibition with IPI-145 has anti-proliferative activity in primary AML cells by inhibiting the activity of AKT and MAPK. Pre-treatment of AML cells with IPI-145 inhibits both adhesion and migration of AML blasts to bone marrow stromal cells. Using shRNA targeted to the individual isoforms we demonstrated that p110δ-knockdown had a more significant anti-proliferative effect on AML cells, whereas targeting p110γ-knockdown significantly inhibited AML migration. The results demonstrate that targeting both PI3Kδ and PI3Kγ to inhibit AML-BMSC interactions provides a biologic rationale for the pre-clinical evaluation of IPI-145 in AML.

## INTRODUCTION

Although clinically, morphologically and biologically acute myeloid leukaemia (AML) appears to represent a heterogeneous group of diseases, the disease seems to rely on common intra-cellular survival and self-renewal pathways downstream of the driver oncogene [1]. It is envisaged that inhibition of common dysregulated signalling pathways in AML would result in novel treatment strategies in the future. Tyrosine kinases (TKs) are an attractive potential target in AML, having been shown to be effective drugable targets in other types of leukaemia [2–4]. Moreover, receptor TKs (RTKs) are mutated in approximately 50% of all patients with AML [5, 6]. Furthermore, cell survival and proliferation pathways dependent on TK activation, including MAPK; phosphoinositide 3-kinase (PI3K)/AKT; mTOR; NF-κB and STATs are deregulated in most, if not all, cases of AML [7–10].

PI3K is an enzyme group that generates phosphatidylinositol 3, 4, 5-triphosphate which provides a membrane docking site for the serine-threonine kinase Protein Kinase B (PKB), also known as AKT [8]. Many of the downstream effects of PI3K are mediated through AKT, which interacts with further downstream signalling molecules, which in turn regulate cell proliferation, cell migration and cell adhesion [8, 11]. It has been observed that activation of the PI3K/AKT pathway is present in over 60% of AML patients and is associated with decreased overall survival [12, 13]. The mechanisms leading to PI3K activation in AML, however presently remain unclear.

Class I PI3K catalytic p110 subunits are divided into 4 isoforms; α, β, δ and γ. Within the p110 catalytic subunit the p110δ isoform is always expressed in AML cells [14, 15]. Conversely the p110γ, p110α and p110β isoforms are heterogeneously expressed [16, 17]. PI3K has previously been reported to be downstream of survival, proliferation, adhesion and migration signals including CD117, FLT3, VLA4 and CXCR4 in a spectrum of benign and malignant haematopoietic cells [11, 18, 19]. In AML these signals play an important role in tumour survival. However, little is known about the role of PI3K in regulating bone marrow-derived AML survival signals.

## RESULTS

### PI3K inhibition reduces the survival of AML blasts

**Figure 1:**
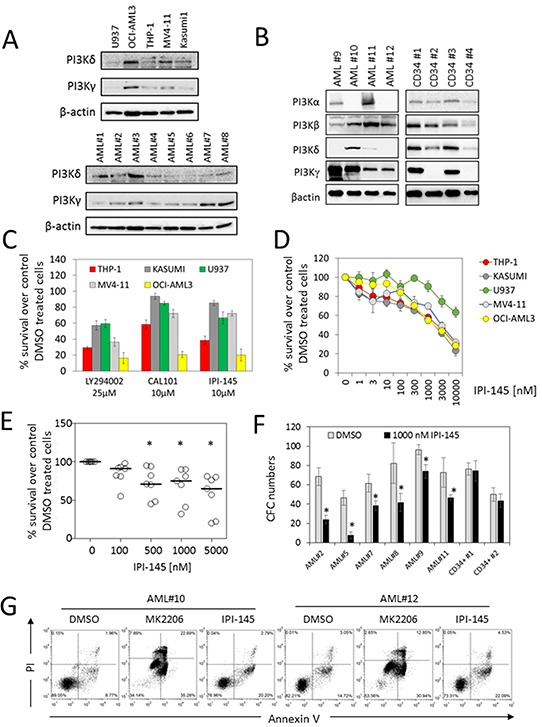
Targeting PI3K inhibits AML survival in AML cell lines and primary AML blasts **A.** AML cell lines and AML patient cells were measured for constitutive levels of PI3Kδ and PI3Kγ by Western blotting, blots reprobed for β-actin to show sample loading. **B.** AML patient cells and CD34+ cells were measured for constitutive levels of PI3Kα, PI3Kβ, PI3Kδ and PI3Kγ by Western blotting, blots reprobed for β-actin to show sample loading. **C.** AML cell lines were treated with 25 μM LY294002, 10μM CAL-101 or 10 μM IPI-145 and cultured for 72 hours and then assessed by CellTiterGlo. **D.** AML cell lines were treated with increasing concentrations of IPI-145 and cultured for 48 hours and then cell viability was assessed by CellTiterGlo. **E.** Primary AML blasts were treated with increasing concentrations of IPI-145 and incubated for 48 hours and then assessed by CellTiterGlo. **F.** AML blasts, AML cell lines and CD34+ control cells were treated with 1000nM of IPI-145 and colony forming assays were performed to show the number of colonies. **G.** Primary AML blasts were treated with MK2206 (1000nM), IPI-145 (1000nM) for 48 hours and then analysed for apoptosis using annexin V and PI staining. * indicates those results which are statistically significant using the Mann-Whitney U test (P-value <0.05), looking at the comparison between the control and treatment groups.

To determine the level of detectable PI3Kδ and PI3Kγ in AML we examined five AML cell lines and eight primary AML samples. Figure 1A shows the expression profile of PI3Kδ and PI3Kγ in AML cell lines and AML primary blasts. Figure 1B shows the expression profile of PI3Kα, PI3Kβ, PI3Kδ and PI3Kγ in normal CD34+ cells and AML primary blasts. However, we observed variations in the level of detection between different cell lines and different primary AML samples: this is thought to be due to high heterogeneity within AML. Next we wanted to examine the effects of targeting PI3Kδ and PI3Kγ in AML. To do this we first assessed the effect of various PI3K inhibitors including LY294002 (pan-PI3K inhibitor, which is not suitable for clinical use as it is toxic in vivo [20]) with a concentration of 25μM [21], CAL-101 (Idelalisib - PI3Kδ inhibitor, which has been found to have a physiologically relevant concentration of between 0.3-1μM [22]) and IPI-145 (PI3Kδ and PI3Kγ inhibitor, which has a physiologically relevant concentration of 1μM [23]) on AML cell lines. We treated AML cell lines with each drug and incubated them for 72hrs. Figure 1C shows that LY294002, IPI-145 and CAL-101 treatment resulted in a decrease in AML cell survival. Next we examined the effect of increasing concentrations of IPI-145 on AML blast survival. Figure 1D shows that all AML cell lines had a dose-dependent reduction in cell survival to increasing concentrations of IPI-145 after culture for 48 hours. Subsequently we examined the effect of IPI-145 treatment on primary AML blasts. Figure 1E shows that primary AML blast survival is reduced in response to increasing concentrations of IPI-145, after culture for 48 hours. Finally we examined the effect of IPI-145 on AML blast colony formation in methylcellulose. AML samples were sensitive to the effects of IPI-145 at concentrations of 1000nM (Figure 1F). Figure 1G shows that the AKT inhibitor, MK2206 and IPI-145 can induce apoptosis in AML cells. Together we report that PI3Kδ and PI3Kγ are constitutively expressed in all AML samples tested and that specific inhibition of these isoforms in AML significantly reduces survival and colony formation. Importantly, this can be achieved at physiologically relevant concentrations of IPI-145 [24, 25].

### IPI-145 inhibits AKT phosphorylation in AML cell lines and primary AML blasts

**Figure 2:**
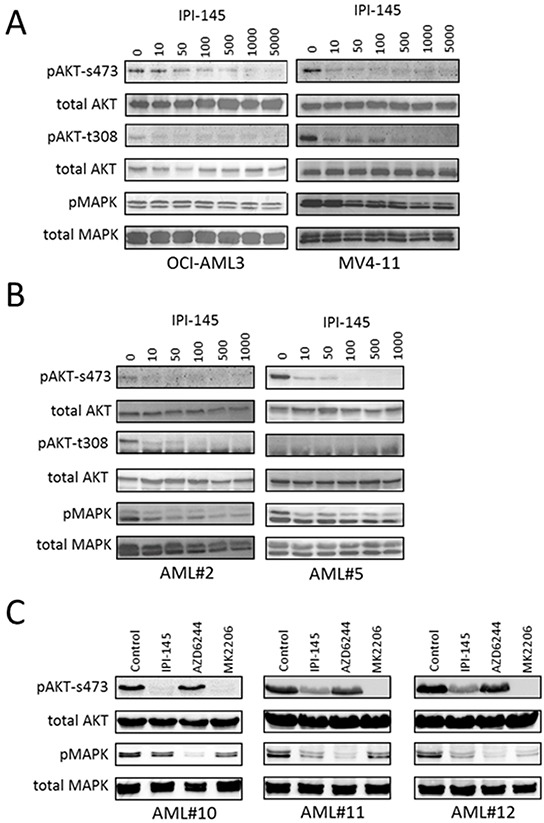
IPI-145 inhibits AKT phosphorylation in AML **A.** and **B.** AML cell lines and primary AML blasts were treated with increasing doses of IPI-145 (nM) and cultured for 4 hours. Whole cell extracts were prepared and Western blot analysis was conducted for pAKT, pMAPK, and total AKT and MAPK protein levels. **C.** AML blasts were treated with IPI-145 (1000nM), MK2206 (1000nM) and AZD6244 (1000nM) and cultured for 4 hours. Whole cell extracts were prepared and Western blot analysis was conducted for pAKT, pMAPK, and total AKT and MAPK protein levels.

The next step was to determine the effect of PI3Kδ and PI3Kγ inhibition on AKT activation. AML cell lines and primary tissue were treated with increasing concentrations of IPI-145 and cultured for 4 hours before Western blot analysis was performed. PI3Kδ and PI3Kγ inhibition showed a reduction in AKT phosphorylation at serine 473 (s473) in all AML cell lines and primary AML tested. There was also a dose dependent reduction in MAPK phosphorylation in response to IPI-145 in OCI-AML3, MV4-11 cell lines (Figure 2A) and primary AML blasts (AML#2 and AML#5) (Figure 2B). These results demonstrate that PI3Kδ and PI3Kγ inhibition with IPI-145 inhibits downstream signalling through AKT phosphorylation at the s473 and t308 and MAPK phosphorylation sites. One primary sample (AML#5) used in this assay did not express constitutive AKT phosphorylation at t308 so no inhibition of this activity was observed in these cells. Figure 2C shows the effect of IPI-145, MK2206 (AKT inhibitor) and AZD6244 (MAPK inhibitor) on three AML samples.

### IPI-145 inhibits adhesion of AML blasts to primary BMSC

**Figure 3:**
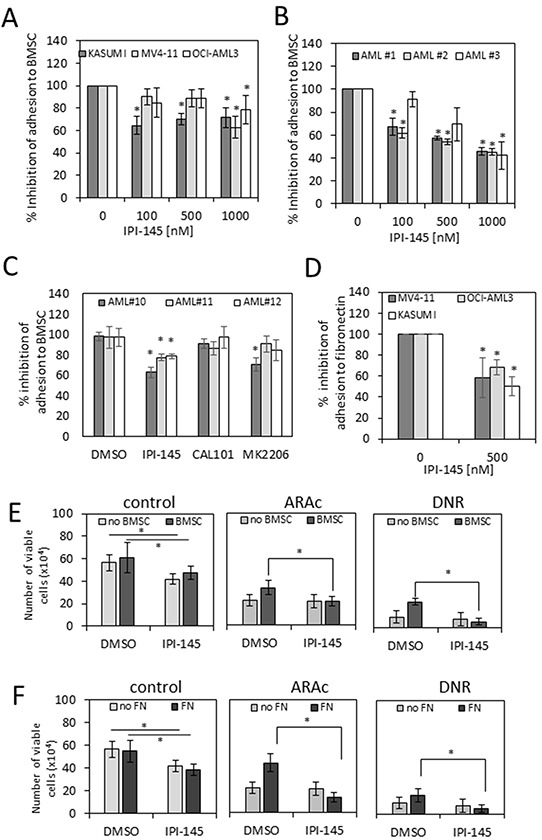
IPI-145 inhibits adhesion of AML blasts to primary BMSC **A.** and **B.** AML cell lines and primary AML blasts (AML#1, AML#2 and AML#3) were treated with increasing doses of IPI-145 and cultured for 16 hours. Calcein-AM was added at 2.5μM for 1 hour, then washed off. The cells were then cultured on top of primary BMSC for 4 hours. Non-adherent cells were taken off with gentle washing. Adherent cells were measured using a fluorescence plate reader. Cell adhesion was then assessed and expressed as percentage of total cells in the assay. **C.** Primary AML were treated with 500nM of IPI-145, 1000nM CAL-101, and 1000nM MK2206. The cells were then cultured on top of primary BMSC for 4h. Non-adherent cells were taken off with gentle washing. Cell adhesion was then assessed and expressed as percentage of total cells in the assay. **D.** AML cell lines were treated with increasing doses of IPI-145 and cultured for 4 hours. Calcein-AM was added at 2.5μM for 1 hour, then washed off. The cells were then cultured on fibronectin (FN) coated plates. Non-adherent cells were taken off with gentle washing. Adherent cells were measured using a fluorescence plate reader. Cell adhesion was then assessed and expressed as percentage of total cells in the assay. **E.** Primary AML blasts were incubated alone (light grey) or on BMSC (dark grey). The samples were then pretreated with DMSO or IPI-145 (500nM) followed by daunorubicin (100nM) (DNR) or cytarabine (500nM) (ARA-c). Live AML blasts cells were counted using trypan blue exclusion. **F.** Primary AML blasts were incubated alone (light grey) or on fibronectin (FN) (dark grey). The samples were then pretreated with DMSO or IPI-145 (500nM) followed by daunorubicin (100nM) (DNR) or cytarabine (500nM) (ARA-c). Live AML blasts cells were counted using trypan blue exclusion. Significance for **E.** and **F.** between IPI-145 treated and control treated samples when cultured with or without BMSC or FN was determined using the two-way ANOVA with Sidak's post test. Results with P < 0.05 were considered statistically significant (*).

The microenvironment is an essential component underpinning survival of AML blasts. Inhibiting AML blast adhesion to BMSCs via VLA4-VCAM/fibronectin interactions via the PI3K/AKT signalling pathway is associated with improvements in chemotherapy-induced tumour cytotoxicity [26]. Therefore, to determine whether PI3Kδ and PI3Kγ inhibition with IPI-145 could overcome BMSC-AML mediated adhesion, AML cell lines and primary AML blasts were treated with increasing concentrations of IPI-145. Figure 3A and 3B show that PI3Kδ and PI3Kγ inhibition with IPI-145 inhibits AML cell line(s) and primary blast adhesion to the BMSC. Next we examined if IPI-145, CAL101 and MK2206 could disrupt AML binding to BMSC (Figure 3C). Figure 3D shows that binding of three AML cell lines to fibronectin was disrupted by IPI-145. Finally we wanted to determine if IPI-145 could impair AML chemo-resistance derived from the BMSC. Primary AML had a higher viability when incubated on BMSC or fibronectin (Figure 3E and 3F) compared to uncoated plates in the presence of daunorubicin 0.1μM (DNR) and cytarabine 0.5μM (ARA-c), both of which are conventional chemotherapeutic agents used in AML. Pre-treatment of 3 AML samples with IPI-145 (500nM) nullified the difference between AML cells incubated on BMSC or fibronectin and those incubated on uncoated plates (Figure 3E and 3F). These results imply that AML chemotherapy resistance (to daunorubicin and cytarabine) conferred by BMSC is able to be overcome by PI3Kδ and PI3Kγ inhibition in combination with these chemotherapy agents.

### IPI-145 inhibits AML migration

**Figure 4:**
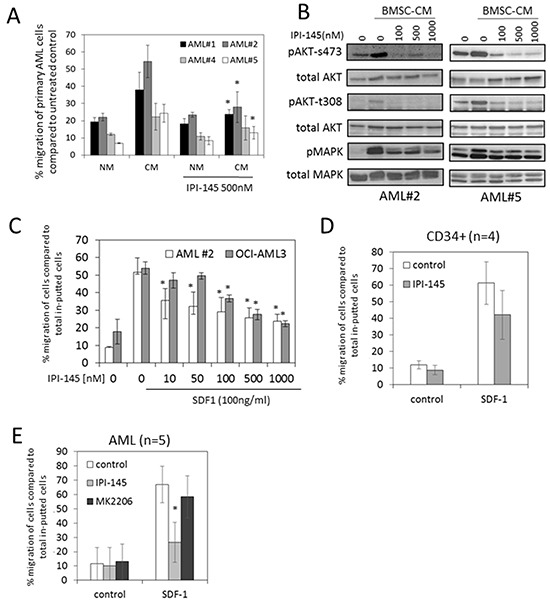
IPI-145 inhibits AML migration to BMSC **A.** Primary AML blasts were pre-treated with IPI-145 and then Calcein-AM was added at 2.5μM for 1 hour, then washed off. The cells were then placed in the upper well of a 5.0μM transwell plate. The lower chamber contained normal media (NM) or BMSC conditioned media (CM). Cells were incubated for 3 hours and then assessed using a fluorescent plate reader. **B.** Primary AML blasts (AML#2 and AML#5) were treated with increasing doses of IPI-145 and cultured for 4 hours. They were then incubated with BMSC conditioned media for 5 minutes. Whole cell extracts were prepared and Western blot analysis was conducted for pAKT (s473 and t308) and total AKT, as well as pMAPK and total MAPK **C.** Primary AML blasts (AML#2) and AML cell line (OCI-AML3) were pre-treated with IPI-145; Calcein-AM was added at 2.5μM for 1 hour, then washed off. The cells were then placed in the upper well of a 5.0μM transwell plate. The lower chamber contained increasing doses of SDF1. Cells were incubated for 3 hours and then assessed using fluorescent plate reader. **D.** CD34+ hematopoietic stem cells were pre-treated with IPI-145 and then Calcein-AM was added at 2.5μM for 1 hour, then washed off. The cells were then placed in the upper well of a 5.0μM transwell plate. The lower chamber contained increasing doses of SDF1. Cells were incubated for 3 hours and then assessed using fluorescent plate reader. **E.** Primary AML blasts were pre-treated with IPI-145 (1000nM) or MK2206 (1000nM) for 30 mins and then Calcein-AM was added at 2.5μM for 1 hour, then washed off. The cells were then placed in the upper well of a 5.0μM transwell plate. The lower chamber contained increasing doses of SDF1. Cells were incubated for 3 hours and then assessed using fluorescent plate reader. For all results in this figure except (B(a two-way ANOVA with Sidak's post test was used. Results with P < 0.05 were considered statistically significant (*).

In order to determine whether PI3Kδ and PI3Kγ inhibition can disrupt AML migration we undertook assays using BMSC conditioned media (CM) as a migration stimulus and normal media (NM) as a control. Figure 4A shows that primary AML blasts migrate towards BMSC CM, which is inhibited by the addition of 500nM IPI-145, which is consistent with the concentrations we have used throughout this study. PI3Kγ has been reported to regulate migration signals via G-protein coupled receptor (GPCR) activation in monocytes and macrophages [27]. In addition, GPCR activation has been shown to signal via the phosphorylation of AKT [28]. Therefore we examined the effect of IPI-145 on AKT s473 and t308 phosphorylation in AML blasts in response to BMSC conditioned media (CM). Figure 4B shows that BMSC CM induced pAKT (s473 and t308) activation is inhibited by IPI-145 at 100nM. Since SDF1-CXCR4 axis has been described as an important mediator of AML migration [29], we analysed the effect of increasing concentrations of IPI-145 treatment on SDF1 induced AML migration. Figure 4C shows that IPI-145 inhibits SDF1 induced AML migration in a dose dependent manner. Figure 4D shows that IPI-145 did not inhibit SDF1-induced CD34+ migration. Figure 4E shows that the AKT inhibitor MK2206 had no effect on SDF1-induced AML blast migration. These results show that PI3Kδ and PI3Kγ regulate AML cellular migration in response to factors secreted by BMSC and furthermore that blast migration can be blocked by IPI-145 through its inhibitory effect on phosphorylation of AKT at t308.

### PI3Kδ modulates adhesion and proliferation and PI3Kγ modulates adhesion, proliferation and migration in human AML

**Figure 5:**
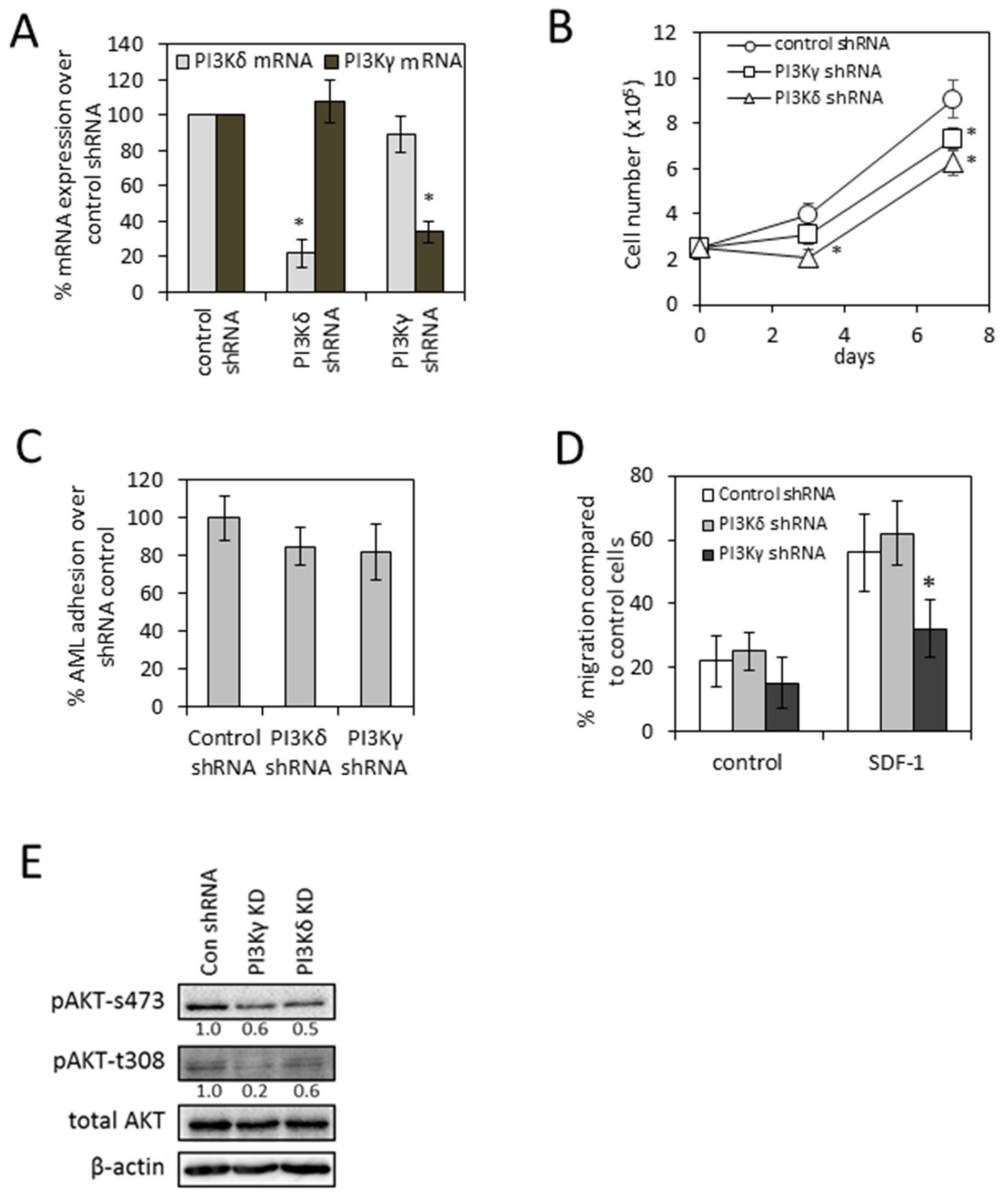
shRNA knockdown of PI3Kδ and PI3Kγ **A.** AML cell line (OCI-AML3) was transduced with either PI3Kδ or PI3Kγ-targeted shRNA or a negative-targeted shRNA lentiviral construct for 72 h. RNA was extracted and assessed for PI3Kδ and PI3Kγ mRNA by real-time PCR. mRNA expression was normalized to GAPDH mRNA levels (n=4). **B.** OCI-AML3 was transduced with either PI3Kδ or PI3Kγ-targeted shRNA lentivirus for up to 7 days. Cell numbers were measured and expressed as a percentage of control transfected cells. **C.** OCI-AML3 was transduced with either PI3Kδ or PI3Kγ-targeted shRNA lentivirus for 72 h. Calcein-AM was added at 2.5μM for 1 hour, then washed off. The cells were then cultured on top of primary BMSC for 4 hours. Non-adherent cells were removed with gentle washing. Adherent cells were measured using fluorescence plate reader. Cell adhesion was then assessed and expressed as percentage of total cells in the assay. **D.** OCI-AML3 were transduced with either PI3Kδ or PI3Kγ-targeted shRNA lentivirus for 72 h and then placed in the upper well of a 5.0μM transwell plate. The lower chamber contained 500ul of serum free media supplemented with SDF1 (100 ng/ml) for 3 hours; we then assessed cell numbers using a flow cytometer. Data were normalised to control-KD cells. **E.** OCI-AML3 were transduced with either PI3Kδ or PI3Kγ-targeted shRNA lentivirus for 72 h and then protein was extracted and analysed for pAKT (s473 and t308) and total AKT using Western blotting. * indicates those results which are statistically significant (P-value <0.05), comparing the KD samples to the control KD.

We next evaluated the genetic inhibition of PI3Kδ and PI3Kγ using shRNA in OCI-AML3. To do this we generated lentivirus-mediated PI3Kδ and PI3Kγ knockdowns. These constructs induced PI3Kδ and PI3Kγ knockdown confirmed for up to 7 days by real-time PCR (Figure 5A). Using these constructs the role of PI3Kδ and PI3Kγ in cell viability was assessed. The introduction of PI3Kδ shRNA inhibited the proliferation of OCI-AML3 at three and seven days post infection (Figure 5B), while the introduction of PI3Kγ shRNA inhibited the proliferation of OCI-AML3 only seven days post infection (Figure 5B). CellTiterGlo was also used to determine cell viability of OCI-AML3 transduced with either PI3Kδ or PI3Kγ knockdown and showed a similar result to the cell count data (data not shown). When we analysed the role of PI3Kδ and PI3Kγ in AML adhesion assays we also found that both isoforms did not inhibit AML adhesion to BMSC (Figure 5C). Next we examined migration and found that only PI3Kγ shRNA inhibited the migration of AML to SDF-1 (Figure 5D). Finally we assessed the effect of PI3Kδ and PI3Kγ inhibition on AKT activity. Both PI3Kδ and PI3Kγ shRNA inhibited AKT-s473, whilst PI3Kγ shRNA could also inhibit t308 phosphorylation (Figure 5E). Together these studies define distinct functions of PI3Kδ and PI3Kγ isoforms in AML.

## DISCUSSION

Despite the recognition that AML represents a morphologically and genetically diverse collection of tumours it is also apparent that these tumours rely on common programs of self-renewal. The PI3K/AKT pathway is known to be constitutively active in 50-80% of patients with AML and is associated with overall decreased survival [15, 30]. Accordingly the PI3K/AKT pathway may represent a valid therapeutic target which could prove applicable and effective in broad populations of patients with AML.

It has been reported that AML proliferation and survival can be inhibited by the pan PI3K inhibitor LY 294002 [13]. Moreover, reports show that idelalisib (PI3Kδ inhibitor) shows little or no inhibitory effect on AML [31]. This suggests that PI3K p110 driven pro-survival signalling is isoform specific. In this study we observe that both PI3Kδ and PI3Kγ are ubiquitously expressed in AML cell lines and AML blasts. We further demonstrate that inhibiting PI3K with LY294002 reduces the survival of all AML cell lines tested, however LY294002 is not suitable for use as a clinical drug due to its high levels of toxicity in-vivo. Moreover, inhibiting both PI3Kδ and PI3Kγ using IPI-145 demonstrates a greater inhibitory effect in AML cell lines compared to inhibiting PI3Kδ alone. Furthermore, when we examine the effect of IPI-145 on primary AML blasts we confirm that we can inhibit survival and colony formation at doses that are reportedly achievable in vivo [24, 25].

In non-malignant myeloid cells both PI3Kδ and PI3Kγ have distinct roles. For example, in mast cells PI3Kδ is required for activation by SCF and IL-3, which induces mast cell proliferation and adhesion [32], while PI3Kγ activation controls chemokine mediated GPCR activation and as such is an essential amplifier of mast cell function [33]. In AML, the mechanisms leading to PI3K/AKT activation are not yet clear. It is known that activation of receptor tyrosine kinases (RTK) expressed on the cell surface of AML have been shown to lead to downstream AKT phosphorylation [18, 19, 34]. Moreover, others have shown that activation of GPCR on AML lead to downstream AKT phosphorylation [35, 36]. What has not been described is the specific PI3K isoform/s that are responsible for the different models of activation of either RTK or GPCR. In addition, there is no data describing which AKT phosphorylation sites are activated by RTK or GPCR in AML. Here using shRNA knockdown and IPI-145 we show that PI3Kδ and PI3Kγ together control the proliferation signals in human AML, whereas PI3Kγ alone controls the GPCR regulated migration signalling.

AKT phosphorylation on s473 can be detected in up to 80% of AML patients while the information on AKT phosphorylation on t308 is less well characterised. A study by Gallay et al (2009), showed that 24 out of 53 AML patients had high AKT phosphorylation on t308 and this (but not AKT phosphorylation on s473) was associated with high-risk cytogenetics and predicted poor overall survival in AML [37]. Our results show that IPI-145 can inhibit constitutive AKT phosphorylation on both s473 and t308. Moreover we also show that conditioned media from BMSC induced AKT phosphorylation on both s473 and t308 which was inhibited by IPI-145. These data demonstrate that in-vitro IPI-145 can inhibit BMSC-mediated AKT phosphorylation at both s473 and t308 in primary AML blasts.

The interactions between tumour and its bone marrow microenvironment have an important role in regulating the survival and proliferation of AML blasts. We have shown that inhibition of PI3Kδ and PI3Kγ can disrupt adhesion of AML blasts to BMSC and fibronectin, and that pre-treatment with IPI-145 can overcome the chemotherapy resistance which BMSC have been shown to provide to AML blasts. Overall we have shown an important role for PI3K and downstream signalling in the AML / BMSC pro-survival interaction. As such we provide data that support the clinical evaluation of combined PI3Kγ and δ inhibition (with IPI-145) in human AML.

## MATERIALS AND METHODS

### Materials

Anti-phosphorylated and pan, AKT, and MAPK antibodies, as well as PI3Kγ were purchased from Cell Signalling Technology (Cambridge, MA, USA). Anti-CD34-PE, anti-CD90-FITC, anti-CD73-PE, anti-CD105-APC antibodies as well as SDF-1 were purchased from Miltenyi Biotec (Auburn, CA, USA). Anti-PI3Kδ antibody was purchased from R&D systems (Abingdon, UK). LY294002, CAL101, IPI-145, MK2206 and AZD6224 were obtained from Selleck Chemicals (Tx, USA). All other reagents were obtained from Sigma-Aldrich (St Louis, MO, USA), unless indicated.

### Methods

#### Cell lines and primary cells

The AML-derived cell lines were obtained from the European Collection of Cell Cultures and DMSZ where they are authenticated by DNA-fingerprinting. In the laboratory they are used at low passage number for a maximum of six months post-resuscitation, testing regularly for Mycoplasma infection. AML blasts were obtained from patients' bone marrow or blood following informed consent and under approval from the UK National Research Ethics Service (LRECref07/H0310/146) Table 1. For primary cell isolation, heparinized blood was collected from volunteers and human peripheral blood mononuclear cells (PBMCs) isolated by Histopaque (Sigma-Aldrich) density gradient centrifugation. AML samples that were less than 80% blasts were purified using the CD34 positive selection kit.

**Table 1:** AML Sample information

ID	Age	Sex	WHO classification	Cytogenetics
AML #1	70	m	AML with minimal differentiation	Normal
AML #2	40	m	AML with minimal differentiation	Normal
AML #3	91	f	AML NOS	Not available
AML #4	59	f	AML with t(8;21)(q22;q22); RUNX1-RUNX1T1	t(8;21)
AML #5	51	m	AML with monocytic differentiation	pending
AML #6	65	m	AML with minimal differentiation	Normal
AML #7	23	f	AML without maturation	t(5;12)
AML #8	65	m	AML with maturation	Trisomy 13
AML #9	37	m	AML without maturation	Normal
AML #10	65	m	AML with maturation	Not available
AML #11	68	m	Therapy related AML	Not available
AML#12	63	m	AML with MDS related changes	Not available

Human bone marrow stromal cells (BMSC) were isolated by bone marrow aspirates from AML patients. Mononuclear cells were collected by gradient centrifugation and plated in growth medium containing DMEM and 20% FBS and 1% l-glutamine. The non-adherent cells were removed after 2 days. When 60%-80% confluent, adherent cells were trypsinised and expanded for 3-5 weeks. BMSCs were checked for positive expression of CD105, CD73, and CD90 and the lack of expression of CD45 and CD34 by flow cytometry as previously described [38].

#### Western immunoblotting

Sodium dodecyl sulfate-polyacrylamide gel electrophoresis and Western blot analyses were performed as described previously. Briefly, whole cell lysates were extracted and sodium dodecyl sulfate-polyacrylamide gel electrophoresis separation performed [39]. Protein was transferred to nitrocellulose and Western blot analysis performed with the indicated antisera according to their manufacturer's guidelines.

#### Proliferation and apoptosis

Cells were treated with different doses of IPI-145 then viable numbers measured with Cell TiterGlo (Promega, Southampton, UK). For the AML-BMSC co-cultures AML cell viability was measured using trypan blue exclusion.

#### Clonogenic methylcellulose assays

Control CD34+ HSC and primary AML cells (1×10^3^ to 5×10^4^ cells) were plated in methylcellulose medium (R&D systems) and colonies were visualised, measured and counted after 10 days. For normal CD34+ CFU-GM, CFU-M, CFU-M and BFU-E was measured.

#### Migration assays

Migration assays were performed in triplicate in transwell permeable plates with 5.0μM pores (Neuroprobe, MD, USA). The lower compartment contained 30μL of conditioned media or serum free media supplemented with 100 ng/ml SDF1 and the cells were applied to the upper compartment and allowed to migrate for 3 h. The amount of viable migrated cells was determined by counting using trypan blue exclusion and expressed as a percentage of the input.

#### BMSC/fibronectin-AML cell adhesion assay

BMSCs were grown in 96-well plates. Primary AML cells were incubated with 2.5 μM calcein-AM for 1 h at 37°C and 5% CO2. The fluorescence-labelled AML cells were added into stromal cell coated 96-well plates and incubated for the indicated time points. Non-adherent calcein-labelled cells were removed by gently washing and adherent cells were quantified in a fluorescence multi-well plate reader. For AML blast adhesion onto fibronectin (FN), 96 well plates were coated with 10 mg/ml FN for 1 h before the fluorescence-labelled AML cells were added. Again non-adherent calcein-labelled cells were removed by gently washing and adherent cells were quantitated in a fluorescence multi-well plate reader.

#### RNA extraction and real-time PCR

Total RNA was extracted from 5 × 105 cells using the Nucleic acid Prep Station from Applied Biosystems (Paisley, UK), according to the manufacturer's instructions. Reverse transcription was performed using the RNA polymerase chain reaction (PCR) core kit (Applied Biosystems). Relative quantitative real-time PCR used SYBR green technology (Roche, Burgess Hill, UK) on cDNA generated from the reverse transcription of purified RNA. After pre-amplification (95°C for 2 minutes), the PCRs were amplified for 45 cycles (95°C for 15 seconds and 60°C for 10 seconds and 72°C for 10 seconds) on a 384-well LightCycler 480 (Roche). Each mRNA expression was normalized against GAPDH mRNA expression using the standard curve method.

#### Transfections

OCI-AML3 were plated onto 12 well plates at 5 × 10^4^ cells/well/0.5ml. Cells were infected with lentiviral stocks at an MOI of 15 in the presence of 8 μg/ml Polybrene. Transduced cells were analysed by real-time-PCR (Roche).

#### Statistical analyses

We used the Mann-Whitney U test to compare results in control to treated groups. Results with P < 0.05 were considered statistically significant (*). We also use the Two-way ANOVA with Sidak's post test. Results with P < 0.05 were considered statistically significant (*). Results represent the mean ± SD of 4 independent experiments. For Western blotting, data are representative images of 3 independent experiments. We generated statistics with Graphpad Prism 5 software (Graphpad, San Diego, CA, USA).
